# Microbiome and spatially resolved metabolomics analysis reveal the anticancer role of gut Akkermansia muciniphila by crosstalk with intratumoral microbiota and reprogramming tumoral metabolism in mice

**DOI:** 10.1080/19490976.2023.2166700

**Published:** 2023-02-05

**Authors:** Zhuxian Zhu, Jixu Cai, Weiwei Hou, Ke Xu, Xuxiao Wu, Yuanlin Song, Chunxue Bai, Yin-Yuan Mo, Ziqiang Zhang

**Affiliations:** a Department of Nephrology, Tongji Hospital, Tongji University School of Medicine, Shanghai, China; b Department of Emergency Medicine, Tongji University School of Medicine, Shanghai, China; c Department of Clinical Laboratory, Tongji Hospital, Tongji University School of Medicine, Shanghai, China; d Department of General Medicine, Tongji University School of Medicine, Shanghai, China; e Department of Respiratory and Critical Care Medicine, Zhongshan Hospital, Fudan University, Shanghai, China; f Institute of Clinical Medicine, Zhejiang Provincial People’s Hospital of Hangzhou Medical College, Hangzhou, China; g Department of Infectious Disease, Tongji Hospital, Tongji University School of Medicine, Shanghai, China; h Department of Respiratory and Critical Care Medicine, Tongji Hospital, Tongji University School of Medicine, Shanghai, China

**Keywords:** Gut microbiota, akkermansia muciniphila (akk), intratumoral microbiome, spatially resolved metabolomics, metabolism reprogramming, crosstalk

## Abstract

Although gut microbiota has been linked to cancer, little is known about the crosstalk between gut- and intratumoral-microbiomes. The goal of this study was to determine whether gut Akkermansia muciniphila (Akk) is involved in the regulation of intratumoral microbiome and metabolic contexture, leading to an anticancer effect on lung cancer. We evaluated the effects of gut endogenous or gavaged exogenous Akk on the tumorigenesis using the Lewis lung cancer mouse model. Feces, blood, and tumor tissue samples were collected for 16S rDNA sequencing. We then conducted spatially resolved metabolomics profiling to discover cancer metabolites in situ directly and to characterize the overall Akk-regulated metabolic features, followed by the correlation analysis of intratumoral bacteria with metabolic network. Our results showed that both endogenous and exogenous gavaged Akk significantly inhibited tumorigenesis. Moreover, we detected increased Akk abundance in blood circulation or tumor tissue by 16S rDNA sequencing in the Akk gavaged mice, compared with the control mice. Of great interest, gavaged Akk may migrate into tumor tissue and influence the composition of intratumoral microbiome. Spatially resolved metabolomics analysis revealed that the gut-derived Akk was able to regulate tumor metabolic pathways, from metabolites to enzymes. Finally, our study identified a significant correlation between the gut Akk-regulated intratumoral bacteria and metabolic network. Together, gut-derived Akk may migrate into blood circulation, and subsequently colonize into lung cancer tissue, which contributes to the suppression of tumorigenesis by influencing tumoral symbiotic microbiome and reprogramming tumoral metabolism, although more studies are needed.

## Introduction

Gut bacteria have been reported to have profound effects on tumor biology, such as influencing tumor treatment response.^1–9^ For example, oral administration of Bifidobacterium can enhance the therapeutic effect of anti-PD-L1, and suppress melanoma proliferation.^9^

Of interest, tumor tissue can harbor a specific microbiota composition, which is defined as intratumoral microbiota.^10–13^ Besides the distinct microbiota composition in different types of tumors, these bacteria can exist in different types of cells, including immune cells.^14–19^ Thus, intratumoral bacteria may play a role in the regulation of cancer progress and treatment. Further characterization of microbiota composition in the tumor context may help to deepen our understanding of microbiota-associated cancer biology.^20^ However, because of its special characteristics and low biomass, the information on the comprehensive characterization of tumoral microbiota is limited,^21^ and the potential effects of intratumoral bacteria remain largely unknown.^20^

A recent study^22^ suggests that Clostridiales members of the gut microbiota may contribute to a lower tumor burden in colorectal cancer (CRC) of mouse models; moreover, its effect depends on the intratumoral infiltration, and subsequent activation of CD8 + T cells. These studies have shed light on the potential strategies of systemic anticancer by modulating gut or intratumoral microbiota.^10^ Strategies to improve therapeutic effects by modulating the microbiota are under intensive investigations. However, little is known about the correlation between gut bacteria and intratumoral microbiota. Especially, it is not fully understood regarding the interaction between the symbiotic microecology and whole-tumor ecosystem.

Studies on Akkermansia muciniphila (Akk) have been attracted much attention. In this regard, Akk exerts a variety of functions and beneficial effects in systemic metabolism, immunity, intestinal barrier, tumor, and other diseases.^5,23–25^ A recent study demonstrated that intestinal Akk can predict clinical response to PD-1 blockade in advanced NSCLC patients,^26^ although the underlying mechanism still remains unclear.^27,28^ Therefore, characterization of tumor metabolism with spatial information, which may be regulated by gut microbiota (such as Akk), will help better understand tumor metabolic reprogramming, and facilitate the discovery of potential metabolites that might be targeted by modulating gut microbiota for anticancer.

However, issues like anticancer effect of Akk on lung cancer, and the role of gut Akk in the regulation of intratumoral metabolic contexture remain unclear. To address these questions, we performed a syngeneic model of lung cancer in mice and focused on dissecting the translocation and influence of gavaged Akk on intratumoral microbiota of lung cancer. Furthermore, we tried to assess the effect of gavaged Akk in affecting cancer metabolic reprogramming by spatially resolved metabolomics analysis at the molecular level.

## Results

### Gut Akkermansia muciniphila inhibits tumorigenesis in Lewis lung cancer mice

**Figure 1.**
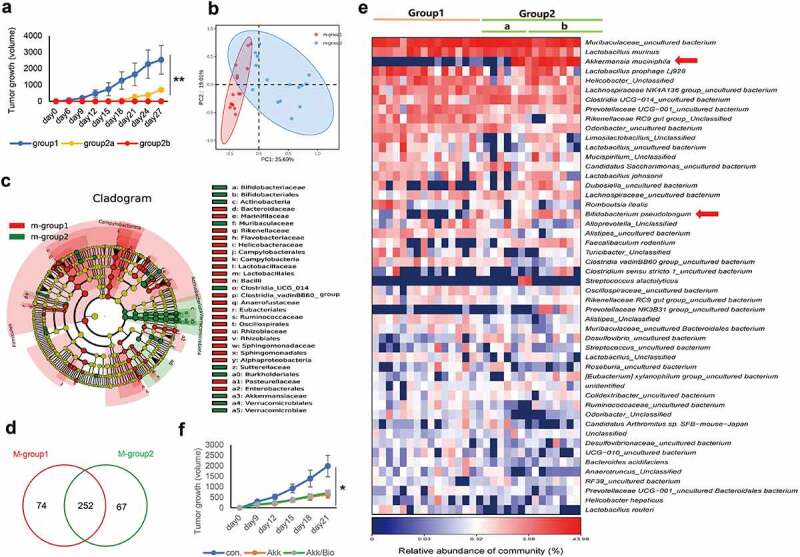
Analysis of gut microbiome community and diversity in lung cancer mouse model. a Identification of three groups after injection of Lewis lung cancer cells based on tumor appearance and growth rate: normal tumorigenesis (group 1), slow tumorigenesis (group 2a) and non-tumorigenesis mice (group 2b). **b** Principal coordinate analysis (PCoA) using weighted-UniFrad of beta diversity. **c** Taxonomic Cladogram from LEfSe, depicting taxonomic association from between microbiome communities from group 1 and group 2. Each node represents a specific taxonomic type. Red nodes denote the taxonomic types with more abundance in group 1 than in group 2, while the green nodes represent the taxonomic types more abundant in group 2. **d** The Venn diagram illustrates the overlapped OTUs between group 1 and group 2. **e** Heatmap of selected most differentially abundant features at the species level. Arrow heads highlighting the taxa enriched in the fecal samples of group 2b (non-tumorigenesis mice). The blue color represents less abundant, red represents the more abundant. **f** Oral administration of Akk or combined with Bifidobacterium pseudolongum influences the outcome of tumorigenesis. Data are shown as mean ± SEM and were analyzed by ordinary one-way ANOVA. *P < .05; **P < .01.

To determine the relationship between endogenous gut microbiota and lung cancer tumorigenesis using lung cancer mouse model, we used a total of 108 mice. Among them, 92 mice had observable tumor nodules (4–5 mm diameter) appeared at 1 week after subcutaneous injection (group 1). The other 16 mice (group 2) were kept under monitoring. Six out of the 16 mice had observable tumor nodules (4–5 mm diameter) appeared at week 2 (group 2a), and the rest (10 mice) did not show any tumor growth even at week 4 after subcutaneous injection (group 2b) (Figure 1a).

To determine differences in the taxonomic composition and microbial diversity of gut microbiota between the two groups (group 1 and group 2), we performed alpha and beta diversity analysis. The 16s rDNA gene sequencing produced a median of 17,080 clean reads from 30 fecal samples. The results of alpha diversity (Chao, Shannon, and Simpson index) were shown in Figure S1a-c. The Operational Taxonomic Unit (OTU) abundance (Chao index), which reflects the species richness, was higher in group 1 than in group 2 (Figure S1a). However, there were no significant difference for Shannon and Simpson index, which reflects the species richness and evenness, between group 1 and group 2 (Figure S1b-c).

Next, we used beta diversity analysis to generate the weighted UniFrac principal coordinates analysis (PCoA) and showed the clustering between group 1 and group 2 (Figure 1b). LEfSe analysis identified a total of 32 discriminative taxa at all taxonomic levels from phylum to genus (LDA>2, p < .05). At the phylum level, the abundance of Verrucomicrobiales, Bifidobacteriaceae, and Actinobacteria were increased in group 2, whereas Bacteroidaceae, Flavobacteriaceae, Helicobacteraceae, and Enterobacterales were enriched in the group 1 (Figure 1c, Figure S1d). Moreover, the Venn diagram detected 326 and 319 OTUs at the species level in the group 1 and group 2, respectively, with 252 OTUs concurrent in the two groups (Figure 1d). At the genus level, the abundance of Akk and Bifidobacterium (Bifidobacterium pseudolongum) were significantly elevated in the group 2 mice, especially in the group 2b mice (Figure 1e). Bar plots of the class taxonomic levels for each fecal samples between the two groups were shown in Figure S2. Of interest, we found that there were significantly elevated gut Akk (belong to Verrucomicrobiales) and Bifidobacterium (Bifidobacterium pseudolongum) in the mice with delayed tumorigenesis (group 2a) or the mice without tumorigenesis (group 2b), compared with regular tumorigenesis mice (group 1).

Thus, we further determined the effect of exogenous gut Akk on tumor proliferation in three groups of mice, including the control group (con.), Akk group and Akk/Bio combined group (mice were gavaged Akk combined with Bifidobacterium pseudolongum). Akk or Bifidobacterium pseudolongum (2 × 10^8^ CFU in 0.15–0.2 ml PBS) was gavaged every 2 days, from week 2 before subcutaneous injection of Lewis cancer cells. The control mice were gavaged with the same volume of PBS, and the mice were kept under monitoring until they were sacrificed. We found that gavaged Akk significantly inhibited tumor proliferation, while there was no significant difference between the Akk and the Akk/Bio group (Figure 1f). Then, we focused on Akk in the following experiments.

### Polar Metabolite profiling with spatially resolved metabolomics through AFADESI-MSI approach

**Figure 2.**
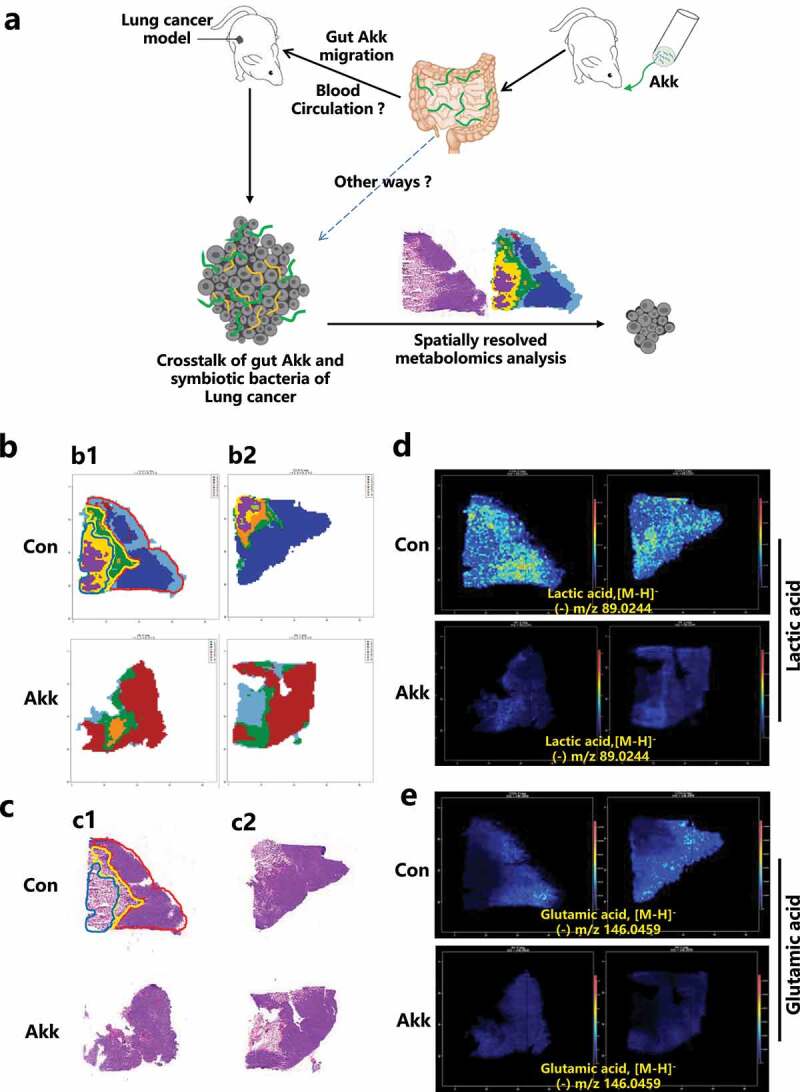
Polar Metabolite profiling in tumor section of lung cancer mouse model. a Figure 2a is the schematic diagram of overall design for this study. **b** Variations in metabolite concentrations in different anatomical cancer subregions, as detected by Clusters of AFADESI-MSI images. c H&E images of cancer tissue section. d Representative MS images of lactic acid (m/z 89.0244) of tumor tissue section. e Representative MS images of Glutamic acid (m/z 146.0459) of tumor tissue section.

Figure 2a is the schematic diagram of overall design for this study. By MSI-based in situ metabolomics combined with metabolic pathway analysis, we identified potential Akk-mediated tumor-associated metabolites and metabolic enzymes in lung cancer tissue. For this analysis, each tumor section was divided into three histologic types based on different anatomical cancer subregions, including the non-necrotic region, para-necrotic region, and necrotic region (Figure 2b,c).

We first performed AFADESI-MSI analysis in the consecutive sections of mouse tumors. The cluster analysis and H&E staining of the tumor sections showed clear substructures of the tumor tissue. We then performed Mapping Polar Metabolites analysis for specific-regional distributions in Lewis lung cancer tissue. Shown in Figure 2b were the clusters of AFADESI-MSI images displaying variations of metabolite concentrations in different anatomical cancer subregions. The H&E staining indicated the clear substructures of different anatomical cancer subregions in the cancer sections (Figure 2c).

Next, tumor tissue sections were scanned by the ESI probe pixel by pixel, and desorbed ions were analyzed both in positive and negative ion modes by a high-resolution mass analyzer.^29^ Specific mass spectra were precisely extracted and illustrated for lactic acid metabolism (Figure 2d) and glutamic acid metabolism (Figure 2e), respectively. These images displayed specific heterogeneous metabolic distributions in the tumor subregions, and they were highly consistent with the functional and structural complexity of the cancer. (Pictures as the representative samples).

### Effects of Akk administration on tumoral metabolism: mapping the region-specific metabolic networks

**Figure 3.**
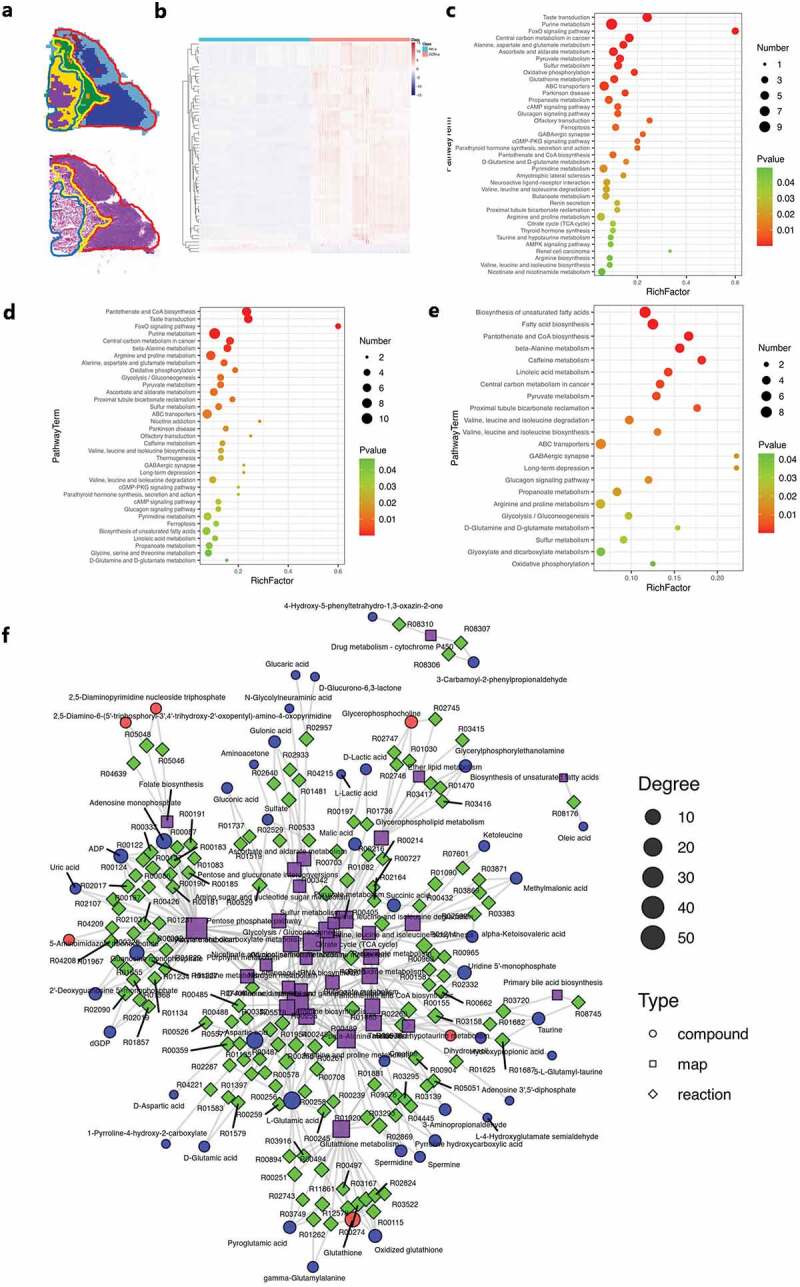
Mapping of the region-specific metabolic networks. a Region-specific MS images of tumor tissue section. MSI, and H&E image. b Heatmap of the metabolites profiling (Desorbed ions analyzed in a positive ion mode). c-e Metabolic network analysis based on the KEGG database, and enriched pathways were displayed by bubble plots, visualized in anatomical cancer subregions, non-necrotic tumor region (c), para-necrotic tumor region (d) and necrotic tumor region (e). f Metabolic networks and contextual pathways acquired by AFADESI-MSI and MetaboAnalyst from the non-necrotic tumor region. Microregional metabolic information were annotated within KEGG pathways.

Microscopic images were then integrated with the matched MS images to form overlaid images with the spatial resolution. Based on the physical structure for extracting the microregional metabolic profile from tumor subregions, we reconstructed a metabolic network. The microscopy-MSI overlaid images revealed that specific mass spectra for each of the three tumor subregions were precisely extracted (Figure 3a-3b and Figure S3a-e) and depicted as metabolites profiling.

The distribution characteristics of the metabolites suggested a variable velocity of metabolic activities in cancer subregions and helped to identify a condition of regional tumor metabolic reprogramming, which can be directly visualized as the metabolic networks of complex metabolic activities regulated by Akk. The region-specific discriminating metabolites were then imported into Kyoto Encyclopedia of Genes and Genomes (www.kegg.jp) to perform metabolic pathway matching analysis and to identify the Akk-regulated metabolic pathways.^30^ Figure 3c–e and Figure S4 summarized the enriched metabolic pathways in the non-necrotic region (Figure 3c, Figure S4a), para-necrotic region (Figure 3d, Figure S4b) and necrotic region (Figure 3e, Figure S4c). Based on metabolites profiling, the enriched pathways were displayed by bubble plots (Figure 3c–e) and bar chart (Figure S4a–c). These results showed that enriched metabolic pathways such as purine metabolism, glutathione metabolism, arginine, and proline metabolism, central carbon metabolism, alanine, aspartate, and glutamate metabolism, pyruvate metabolism, glycolysis/gluconeogenesis, pyrimidine metabolism, and fatty acid biosynthesis in tumor tissue were significantly influenced by the Akk gavage.

This KEGG-based metabolic network allows for a deep understanding of lung cancer metabolic activities at the system level. In this study, microregional metabolic information and their interactive network in the non-necrotic tumor tissue were grouped into 26 KEGG pathways. Further analysis of the contextual pathways showed that there is a close regulatory relationship among a variety of metabolic pathways such as glutamine metabolism, purine and pyrimidine metabolism, glycolytic metabolism, and glutathione metabolism (Figure 3f).

### Effects of Akk administration on tumoral metabolism: Akk regulates glycolytic metabolism

**Figure 4.**
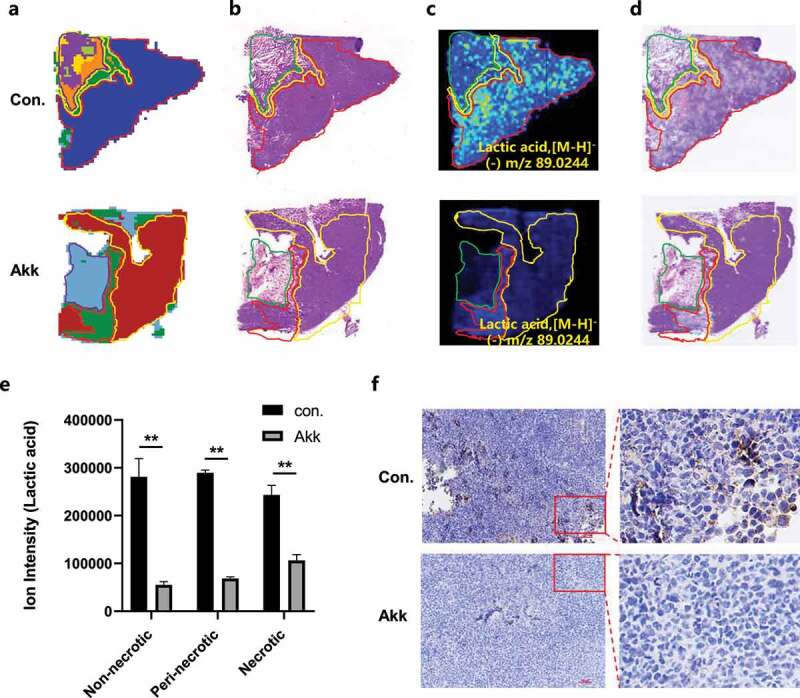
In situ visualization of crucial metabolite and metabolic enzymes in the glycolytic metabolism pathway. a-d Representative MS images of Clusters (a), H&E (b), MSI of lactic acid (c) and overlaid optical and MSI image of lactic acid (d) of Lewis lung cancer tissue. **e** Lactic acid level in the different anatomical cancer subregions, non-necrotic, para-necrotic and necrotic tumor tissue of Lewis lung cancer tissue. **f** IHC staining of the metabolic enzyme LDHA in lung cancer tissue sections between the control and Akk gavaged mice. Data are shown as mean ± SEM and were analyzed by ordinary one-way ANOVA. *P < .05; **P < .01.

In this study, microscopic images and MSI images of metabolites were overlaid according to their physical structure to extract the microregional metabolic profile of lactic acid in different anatomical cancer subregions (Figure 4a-e). It is evident that glycolytic metabolism was dysregulated in lung cancer tissues: the ion intensity of lactic acid was highly expressed in the whole tumor regions. The gavaged Akk dramatically down-regulated the lactic acid level in the different anatomical cancer subregions (Figure 4c).

Consistent with metabolite profiling, IHC revealed that LDHA, as an enzyme regulating glycolytic metabolism, was highly expressed in the cancer region, while it was significantly decreased in the Akk gavaged group (Figure 4f).

### Effects of Akk administration on tumoral metabolism: Akk regulates glutamine metabolism as determined by in situ visualization of crucial metabolites

**Figure 5.**
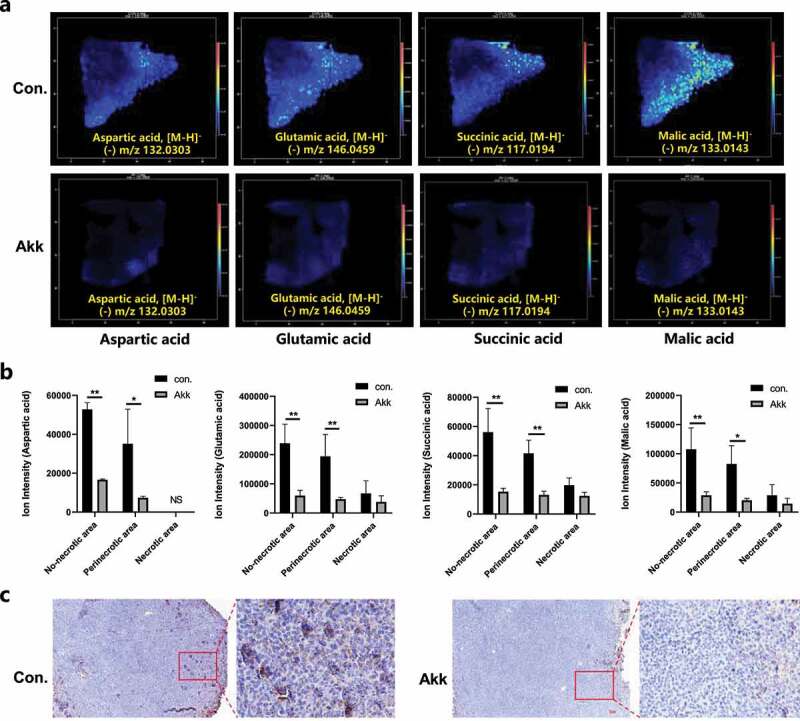
In situ visualization of crucial metabolites and metabolic enzymes in the glutamine metabolism pathway. a Representative MSI images of glutamine metabolism: spatial expressions of Glu, aspartic acid, succinate and malic acid in cancer tissue sections. b Glu, aspartic acid, succinate, and malic acid levels in the different cancer subregions (non-necrotic, para-necrotic, and necrotic tumor), between the control and the Akk gavaged mice. c IHC staining of the metabolic enzymes GLS in Lewis lung cancer tissue section, between the control and the Akk gavaged mice. Data are shown as mean ± SEM and were analyzed by ordinary one-way ANOVA. *P < .05; **P < .01.

The MS image is composed of consecutive pixels, and thus it can reflect the relative content of the metabolites in the tumor, especially for the region-specific discriminating metabolites from the different tumor subregions. Studies have shown that cancer cells display a strong addiction to glutamine (Gln).^31^ The catabolism of Gln is mediated by glutaminase (GLS) through the hydrolysis of Gln to glutamate. Our study using MSI analysis suggested that Gln metabolism was significantly dysregulated in the Lewis lung cancer tissue. As shown in Figure 5a and Figure S5, Glu, aspartic acid, succinate and malic acid as the hydrolysis products of Gln, were dramatically increased in Lewis lung cancer tissues, however, they were significantly down-regulated in the Akk group, as compared with the control.

Moreover, the ion-intensity based MSI offers an approach to the evaluation of the altered intensity across different tissue regions, which may reflect the GLS-mediated in situ glutamate (glutamic acid, Glu) hydrolysis rate. The spatial expressions of Glu, aspartic acid, succinate, and malic acid in the different cancer subregions are shown in Figure 5b. Metabolic enzymes that are directly associated with the altered metabolites in pathways were chosen as potential tumor-associated metabolic enzymes. GLS catalyzes the first reaction in the primary pathway for the catabolism of Gln. IHC staining showed that GLS was remarkably up-regulated in cancer tissue, while it was significantly down-regulated in the Akk group (Figure 5c), which is in good agreement with the intensity-based MS images of Glu.

### Effects of Akk administration on tumoral metabolism: Akk regulates purine and pyrimidine metabolism

**Figure 6.**
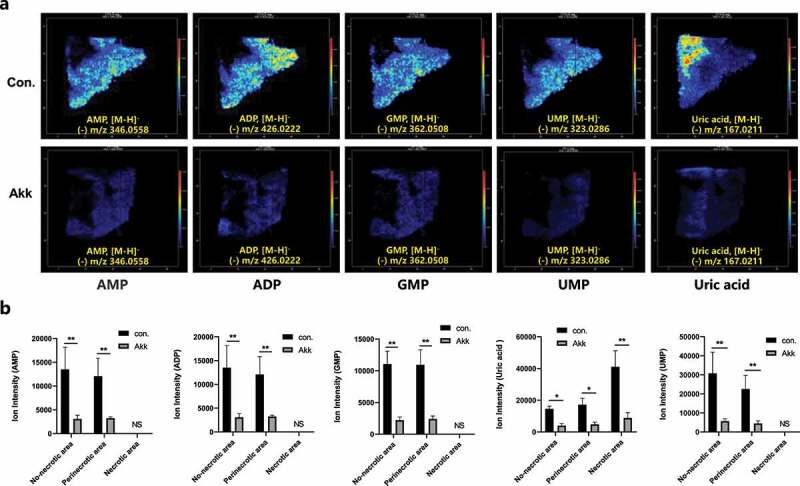
Gavaged Akk downregulates nucleotide biosynthesis metabolism as determined by in situ visualization of crucial metabolites and quantitative analyses. a Representative MS images of AMP, ADP, GMP, UMP and uric acid levels in lung cancer tissue, between control and the Akk gavaged mice. **b** Quantitative analysis of AMP, ADP, GMP, UMP and uric acid levels in the different anatomical cancer subregions, between the control and the Akk gavaged mice. Data are shown as mean ± SEM and were analyzed by ordinary one-way ANOVA. *P < .05; **P < .01.

The synthesis of nucleotides can be regulated by a variety of metabolic pathways, and these nucleotides are essential nutrients for the maintenance of the rapid proliferation of cancer cells. One of the mechanisms is through enhancing glycolytic metabolism or Gln metabolism to produce more molecular precursors required for nucleotide synthesis.^32^ MSI analysis indicated that nucleotide biosynthesis, such as AMP, ADP, GMP, and UMP, were significantly expressed in Lewis lung cancer of the control mice (Figure 6a), meanwhile the nucleotide biosynthesis was dramatically down-regulated in cancer tissue of the Akk group (Figure 6b).

### Influence of gavaged Akk on gut microbiota

**Figure 7.**
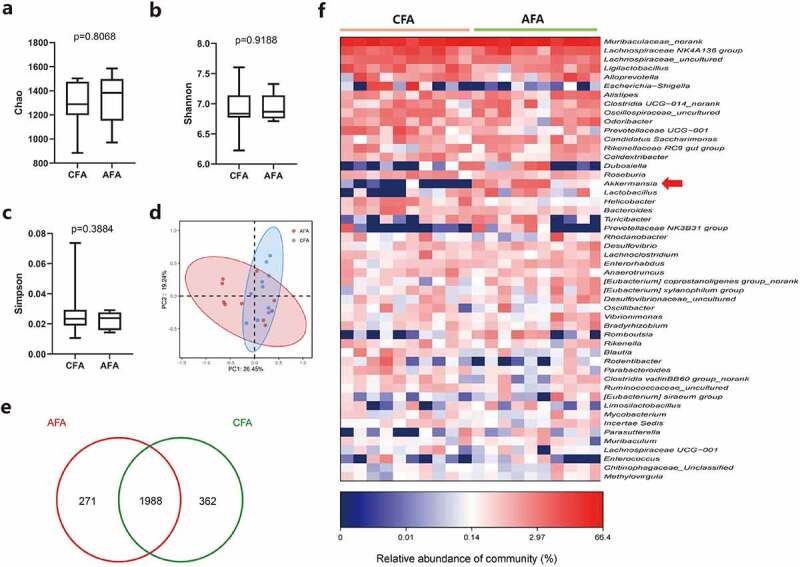
Influences of gavaged Akk on gut microbiota in mice. a-c Alpha diversity box plot (Chao, Shannon and Simpson index) in the control (CFA) and Akk gavaged mice (AFA). d Principal coordinate analysis (PCoA) using weighted-UniFrad of beta diversity. e The Venn diagram illustrates the overlapped OTUs between the control and Akk gavaged mice. f Heatmap of selected most differentially abundant features at the genus level. The blue color represents less abundant, red represents the more abundant (CFA vs AFA). Data are shown as mean ± SEM and were analyzed by t test. *P < .05; **P < .01.

Given the influences of gavaged Akk on gut microbiota, we sought to determine the gut microbiome composition and diversity between the control and Akk gavage group (n = 8). The alpha diversity of the gut microbiota was analyzed by 16S rDNA sequencing, which revealed that there was no significant difference of the species richness index (Chao) (p = .8068), Shannon (p = .9188) or Simpson index (p = .3884) between the control (CFA) and the Akk gavaged mice (AFA) (Figure 7a-c). With beta diversity, the weighted UniFrac principal co-ordinates analysis (PCoA) showed the clustering between control and the Akk mice (Figure 7d). Moreover, as shown in the Venn diagram, 2350 and 2259 OTUs were detected in control and the Akk gavaged mice, respectively, with 1988 OTUs concurrent in the two groups (Figure 7e).

To identify the specific microbial communities associated with Akk, we analyzed the composition of the gut microbiota in the two groups by LEfSe analysis. At the phylum level, Verrucomicrobiales and Saccharimonadia were increased in the feces of Akk gavaged mice (AFA), whereas Proteobacteria, Helicobacteraceae, Bacteroidales, Campylobacter ota, and Pseudomonadales were enriched in the control (CFA) (Figure S6a-b). Bar plots of the class taxonomic levels between the control and the Akk gavaged mice were shown in Figure S6c. At the genus level, the abundance of Akk was found to be elevated in gut microbiota of the Akk-gavaged mice, whereas the abundance of Enterobacterales, Helicobacter, Bacteroidota was increased in gut microbiota of the control mice (Figure 7f).

### Influence of gavaged Akk on tumoral microbiota

**Figure 8.**
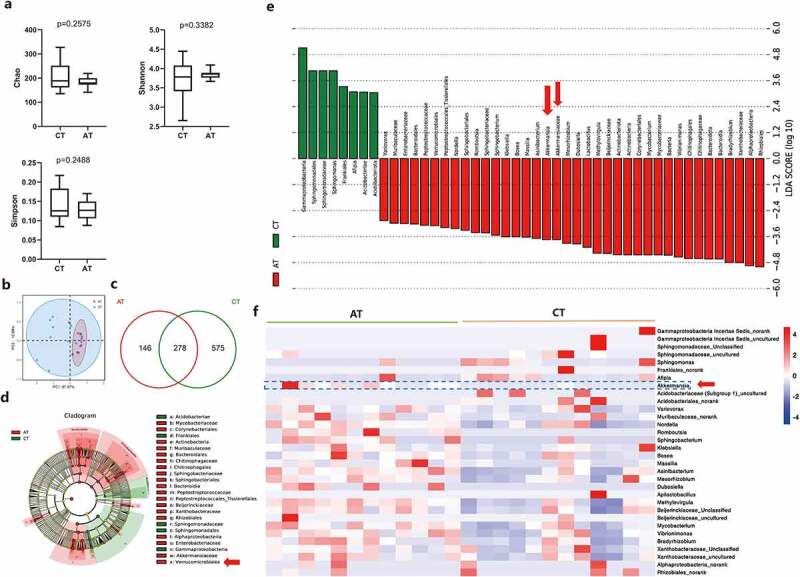
Influences of gavaged Akkermansia muciniphila on intratumoral microbiome in lung cancer mice model. a Alpha diversity box plot (Chao, Shannon and Simpson index) in the control and Akk gavaged mice. (CT vs AT group). **b** Principal coordinate analysis (PCoA) using weighted-UniFrad of beta diversity. **c** The Venn diagram illustrates the overlapped OTUs between the control and Akk gavaged mice. (d-e) Tumor microbiome communities are significantly different between the control and Akk gavaged mice. Taxonomic Cladogram from LEfSe, depicting taxonomic association between microbiome communities from the control and Akk gavaged mice. Each node represents a specific taxonomic type. Red nodes denote the taxonomic types with more abundance in the Akk gavaged mice than the control mice, while the green nodes represent the taxonomic types more abundant in the control. LDA score computed features differentially abundant between the control and Akk gavaged mice. The criteria for feature selection is Log LDA Score > 2. **f** Heatmap of selected most differentially abundant features at the genus level between tumoral microbiome communities from the control (CT) and Akk gavaged mice (AT). The taxa enriched in the lung cancer tissue are highlighted here (Akk). The blue color represents less abundant, red represents the most abundant. Data are shown as mean ± SEM and were analyzed by t test. *P < .05; **P < .01.

We next sought to determine the influences of gavaged AKK on intratumoral microbiota. The alpha diversity of tumoral microbiota was analyzed by 16S rDNA sequencing. There was no difference of the species richness Chao index (p = .2575), Shannon (p = .3382) or Simpson (p = .2488) index between the control (CT group) and the Akk gavaged mice (AT group) (Figure 8a). PCoA analysis for beta diversity showed the clustering between the control and Akk gavaged mice (Figure 8b). Moreover, as shown in the Venn diagram, 853 and 424 OTUs were detected between the control and Akk gavaged mice, respectively, with 278 OTUs concurrent in the two groups (Figure 8c).

LEfSe analysis showed that at the phylum level, the abundance of Bradyrhizobium, Vibrionimonas Verrucomicrobiales, and Lactobacilius was increased in tumors in the Akk gavage group, whereas the abundance of Gammaproteobacteria, Sphingomonadales, and Acidobacteriae was enriched in the control (Figure 8d-e). As expected, the abundance of Akk was elevated in the tumor tissue of the Akk gavaged lung cancer mice, compared with the control (AT vs CT group) (Figure 8d-8f). Furthermore, to compare the microbial composition of different samples, we set up 5 groups: Lewis lung cancer cells before the subcutaneous injection for establishment of lung cancer mice model (Cell), fecal samples from the control mice (CFA), tumor tissue samples from the control mice (CT), fecal samples from the Akk-gavaged mice (AFA), tumor tissue samples from the Akk-gavaged mice (AT). We compared and analyzed the enriched top 100 microbiota among these groups. As shown in Figure S7a unlike the composition of gut microbiota, the predominant microbiota in tumor tissue such as Staphylococcus, Vibrionimonas, Bacillus, and Rhodanobacter were not obvious in the gut.

We further compared the predominant tumoral microbial composition of the control mice (CT) or Akk-gavaged mice (AT) with the Lewis lung cancer cells before subcutaneous injection for establishment of lung cancer mouse model (Cell). In particular, an additional analysis of the intracellular bacteria of Lewis lung cancer cells before being used for establishment of lung cancer mice model (Cell group) were taken as the background microbiota. With this approach, we would be able to determine the changes of intratumoral microbiota after tumorigenesis. As shown in Figure S7b, increased abundance of microbiota in tumor tissue from either the control or Akk-gavaged lung cancer mice, especially the Akk-gavaged mice, Akkermansia, Lactobacillus, Bifidobacterium, Staphylococcus, and Bacteroides (marked with red arrow bar) were significantly increased in the tumor tissue, compared with the Lewis cell samples. On the other hand, these common intestinal microbiota were not detected in the Lewis cell line used for lung cancer mouse model. These results suggest that the tumor microbiota contains higher abundance of Akkermansia and other “gut microbe” and they may derive from the intestinal tract. Bar plots of the class taxonomic levels for each tumor sample between control and the Akk gavaged mice were shown in Figure S7c.

### Crosstalk between gut- and tumoral microbiota: translocation of gut bacteria into lung cancer tissue likely through systemic circulation

The positive relationship between gut Akk and intratumoral microbiota suggests that there may be a potential translocation of gut bacteria into cancer tissue. Thus, we asked whether the gut Akk migrates into tumor tissues through systemic blood circulation.

Under SPF conditions, C57BL/6 mice were divided into two groups, the control (CB) and bacteria gavaged group with Akk and Bifidobacterium (AB). The AB mice were gavaged with 2 × 10^8^ CFU of Akk in 0.15 ml PBS, along with 2 × 10^8^ CFU Bifidobacterium (ProBiota Bifido, SeekingHealth, USA) in 0.15 ml PBS, and were monitored until sacrifice. Blood samples were collected at hour 2, and hour 6 after bacterial gavage for 16S rDNA sequencing analysis. Each blood sequencing sample were mixed with blood collected from three mice.

**Figure 9.**
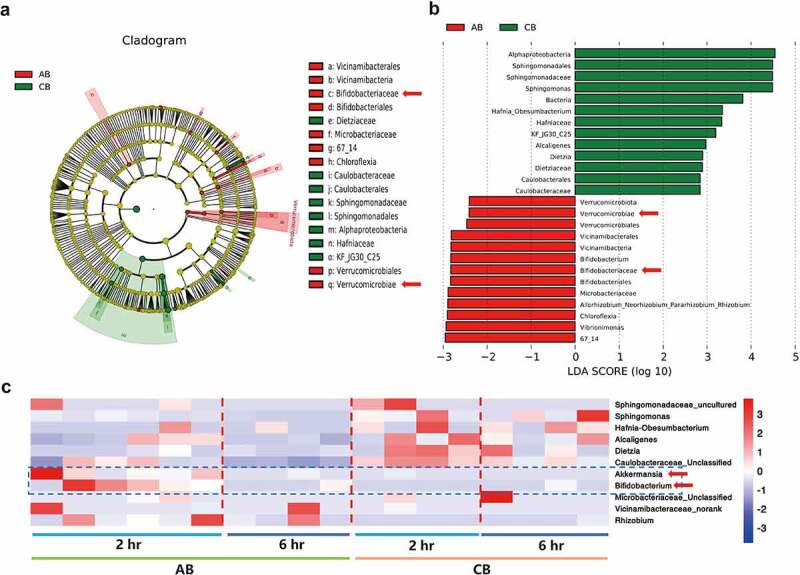
Detection of gut bacteria in blood circulation. a-b Tumor microbiome communities in blood circulation of the control mice (CB) and Akk gavaged mice (AB) (CB vs AB group), blood samples were collected at 2 h and 6 h after bacterial gavage. Taxonomic Cladogram from LEfSe, depicting taxonomic association between microbiome communities from the control and Akk gavaged mice. Each node represents a specific taxonomic type. Red nodes denote the taxonomic types with more abundance in the Akk gavaged mice than the control mice, while the green nodes represent the taxonomic types more abundant in the control. LDA score computed features differentially abundant between the control and Akk gavaged mice. The criteria for feature selection is Log LDA Score > 2. c Detection of bacteria by 16 rDNA sequencing. Heatmap of selected most differentially abundant features at the genus level. The blue color represents less abundant, red represents more abundant. Blood samples were collected at 2 h and 6 h after bacterial gavage.

The alpha diversity of the species richness Chao index (p = .3834), Shannon (p = .1653) or Simpson (p = .073) index between the control and the gavaged mice were showed in Figure S8a. The beta diversity between the control and the Akk gavaged mice, by the weighted UniFrac principal co-ordinates analysis (PCoA), was shown in Figure S8b. To identify the specific microbial communities associated with Akk and Bifidobacterium gavage, we analyzed the composition of the microbiota in blood samples using LEfSE approach. At the phylum level, the abundance of Verrucomicrobia (Akk belongs to Verrucomicrobia) and Bifidobacteriaceae were increased in blood samples of the bacteria gavaged mice (Figure 9a,b). At the genus level, Akk and Bifidobacterium were found to be elevated in blood at hour 2 after bacteria gavage, while Akk or Bifidobacterium could not be obviously detected at hour 6 after bacteria gavage (Figure 9c). The difference of microbiota communities in blood between the control (CB) and the bacteria gavaged mice (AB) was shown with bar plots at the class taxonomic levels (Figure S8c). Both Bifidobacterium and Akk were detected in the blood samples.

### Association between the differential tumoral microbiota and discriminative metabolites

**Figure 10.**
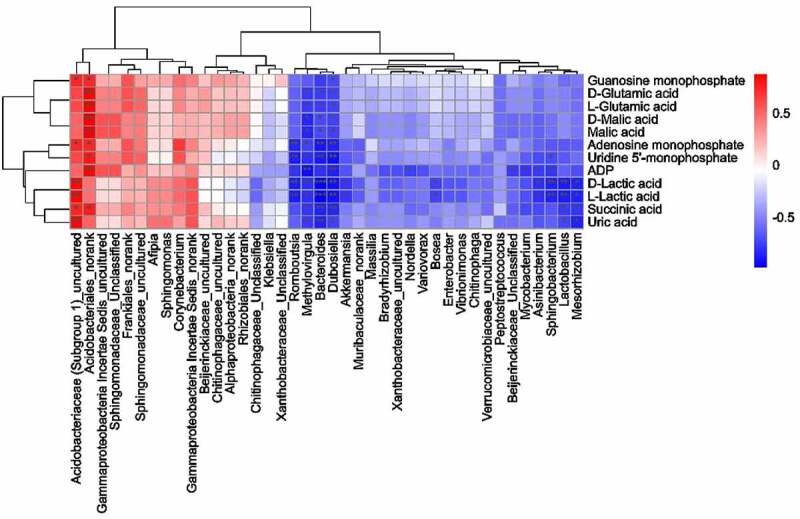
Association analysis between the discriminative bacteria and differential metabolites. Spearman’s correlation analysis shows the association between discriminative genus and differential metabolites based on the taxonomic analysis of LEfSe. Red, positive correlations; blue, negative correlations.

We assessed the association between the differential genus and metabolites in tumor tissue by the Spearman’s correlation analysis. Figure 10a shows the correlation of the top differential genus based on the taxonomic analysis of LEfSe and the top discriminative metabolites in the pathways such as glycolytic metabolism, Gln metabolism, purine, and pyrimidine metabolism.

In addition to Akk, we also found that Asinibacterium, Lactobacillus, Bacteroides, Dubosiella, Methylovirgula, Romboutsia, Mesorhizobium, Sphingobacterium, Mesorhizobium, and Bosea were enriched in tumor tissue of Akk gavaged lung cancer mice, and they exhibited a significantly negative correlation. Acidobacteriaceae were enriched in tumor tissue of the control mice, which exhibited a significantly positive correlation with the differential metabolite (lactic acid) in the pathway of glycolytic metabolism in non-necrotic tumor tissue (Figure 4 and Figure 10a).

Moreover, Bacteroides, Dubosiella, and Methylovirgula enriched in tumor tissue of Akk gavaged lung cancer mice exhibited a significantly negative correlation, while Acidobacteriales or Acidobacteriaceae enriched in tumor tissue of the control mice exhibited a significantly positive correlation with the differential metabolites in the pathway of Gln (glutamic acid, succinic acid, and malic acid) and adenosine metabolism (AMP, ADP, UMP, GMP, and uric acid) in non-necrotic tumor tissue. These results indicate that these Akk-influenced symbiotic bacteria likely play a regulatory role in the Gln and adenosine metabolism. Moreover, Akk-influenced symbiotic bacteria exhibited significant correlations with discriminative metabolites in the respective pathways of metabolic network in Lewis lung cancer tissue, as shown for the top 100 differential genus and the discriminative metabolites (Figure S9).

## Discussion

Recent evidence indicates that gut bacteria may play a regulatory role in other locations beyond the gut.^13^ However, little is known about the crosstalk of gut bacteria and the tumoral microbiota. The present study represents the first report to explore the influence of gut microbiota (e.g., Akk) on the intratumoral microbiome and metabolic network by the Lewis lung cancer mouse model. Of great interest, both endogenous and exogenous Akk could exert a favorable anticancer effect, supporting the notion that gut microbiota can shape the tumor microenvironment and cancer progression. Akk gavaged mice reveal a distinctive tumor microbiome signature with specific bacterial genus, and thus this approach may provide a base for future exploration of novel therapeutic strategies.

A recent study demonstrated that intratumor bacteria promote lung metastasis without affecting primary tumor growth in breast cancer.^33^ The intratumor bacteria can enhance resistance to fluid shear stress by reorganizing actin cytoskeleton. Unlike these harmful bacteria, we found that gavaged Akk have a beneficial effect by suppressing tumor growth in lung cancer mice.

As an antitumor strategy, oral administration of commensal bacteria has attracted much attention.^3,4,34^ For example, oral administration of Bifidobacteria has been proven to have an antitumor effect. Furthermore, gavaged Bifidobacterium could enhance the anticancer efficiency of immunotherapy by colonizing into tumor tissue.^35^ Other bacteria, such as Akkermansia and Faecalibacterium, also have been suggested to enhance the therapeutic effect of ICI.^36–39^ However, it is still unknown whether and how they can localize into tumor tissue and contribute to the antitumor function.

Most bacterial communities found in tumor tissue commonly exist in the gut microbiota,^40,41^ suggesting a potentially translocation of bacteria from gut into the cancer tissue. Our finding is consistent with the report that gut microbiota has the capacity to colonize into pancreatic tumors and this colonization can modify the overall tumoral microbiota in pancreatic ductal adenocarcinoma (PDAC) patients.^13^ However, different from these studies, we demonstrate that this colonization by Akk can modify the overall intratumoral microbiota of lung cancer. In particular, we identify a signature of tumor bacterial taxa: Akkermansia, Asinibacterium, Lactobacillus, Bacteroides, Dubosiella, Methylovirgula, Romboutsia, Mesorhizobium, Sphingobacterium, Bradyrhizobium, Vibrionimonas, Bosea, and Enterobacteriaceae are significantly increased in the lung cancer tissue of Akk gavaged mice, whereas Acidobacteriales or Acidobacteriaceae are significantly increased in the control mice.

Given that Akk or Bifidobacterium, as anaerobic bacterium residing in gut, which can colonize inside tumor, they may be candidate bacteria for clinical targeting for tumor prevention or treatment. The crosstalk between gut bacteria (Akk) and the tumor microbiota may have a powerful impact in determining the lung cancer progression.

The interactions between probiotics and human body are complex. Gut microbiota achieves a dynamic balance through mutual competition and cooperation.^42^ A recent study has shown that administration of Akk can improve the clinical efficacy of PD-1 anti-cancer treatment in NSCLC patients, and the gut Akk abundance is related to the better prognosis. However, excessive Akk is also associated with poor prognosis. For example, the median overall survival time (OS) in the “low Akk“ group was significantly longer than that of the “high Akk“ group or the “no Akk” group.^26^ Consistently, our results show that gavaged Akk combined with Bifidobacterium pseudolongum does not increase the anti-cancer effect, compared with Akk alone, which may reflect the potential complex interactions among different strains of gut microbiota.

Although alterations of microbial diversity in lung cancer tissue due to gavaged Akk may have an immunoregulatory impact, its role in the antitumor response is not entirely clear. Notably, our results provide evidence for the first time that gut Akk influences cancer microbiota diversity and composition, and ultimately influence the tumor metabolic reprogramming. Thus, the microbiota may exert anticancer effects through regulation of cancer metabolism. However, a complex interplay exists among the gut- or tumor-microbiota, tumor metabolic microenvironment, and the precise mechanisms of which and how microbes modulate what kind of metabolic pathways warrant further investigation.

Tumor tissue contains global biological metabolic information at both metabolite and the metabolic enzyme levels. MSI data combined with OPLS-DA analysis enable the discovery of region-specific discriminating metabolites. Moreover, as the important nodes in the network, metabolic enzymes regulate complex metabolic reactions and have been considered as the potential anticancer targets. Thus, the tumor-associated metabolic enzymes and corresponding metabolites information expand our understanding of the complex tumor metabolic reprogramming. Our results demonstrate that differentially expressed metabolites and metabolic enzymes closely associated with tumor growth can be identified by region-specific and pathway-related metabolites through the spatially resolved metabolomics approach. We also paid more attention to key enzymes that are directly related to the dysregulated metabolites. However, the potentially altered metabolic enzymes based on spatially resolved metabolomics still need subsequent IHC validation.^43^

MSI-based in situ metabolomics combined with metabolic pathway analysis (e.g., KEGG) help to uncover potential tumor-associated and Akk-regulated glycolytic metabolism and metabolic enzymes in lung cancer tissue. Our results suggest that the purine metabolism, glutathione metabolism, arginine and proline metabolism, central carbon metabolism in cancer, alanine, aspartate, and glutamate metabolism, pyruvate metabolism, glycolysis/gluconeogenesis, pyrimidine metabolism, and fatty acid biosynthesis are significantly altered due to the oral administration of Akk.

Under the condition of sufficient oxygen, normal cells gain energy mainly through oxidative phosphorylation, while tumor cells mainly rely on glycolysis metabolism. Lactic acid, an important product of tumor glycolysis metabolism, is involved in the regulation of tumor progression. LDHA is the key enzyme of glycolysis metabolism, which catalyzes the production of L-lactic acid from pyruvate, and is regarded as one of the potential tumor-associated metabolic enzymes in lung cancer.^44^ Our results suggest that Akk regulates metabolic enzymes, such as LDH and GLS, which are validated by IHC, and these enzymes play a critical role in lung cancer. Gln is necessary for the maintenance of cell energy metabolism, nucleotide, and amino acid biosynthesis. Glutamate (glutamic acid, Glu) is one of the major nutrients consumed by tumors. Glutaminolysis refers to the hydrolysis of glutamine into glutamic acid, aspartic acid, pyruvic acid, lactic acid, alanine, citric acid, etc. Moreover, cancer cells often elevate the rate of nucleotide biosynthesis and metabolism reprograming to meet the need for high cell proliferation. As one of the most important metabolic components, purine nucleotides and pyrimidine nucleotides are essential for tumorigenesis. For example, uridine, an important nucleoside precursor for the synthesis of RNA, is also involved in the regulation of purine nucleotide biosynthesis or carbohydrate metabolism.^45^ Disturbance of tumor metabolism can provide more biological macromolecular precursors or coenzymes for the nucleotide synthesis. Thus, this spatially resolved tumor metabolic information in lung cancer mouse model offers insights into gut Akk-mediated complex cancer metabolic reprogramming.

The complex interactions between gut microbiota and other organs will affect the body functions, forming the “gut organ axis”.^46^ How systemic infection in the lungs can produce a change in the gut microbiota or how gut microbiota affects lung cancer? It is possible that systemic pathological changes, including immune cell function, and disturbance of cytokine and inflammatory medium secretion, may affect the intestinal flora by disturbing the normal mucosal habitat or vice versa.

Moreover, our results suggest that gut microbes such as Bifidobacterium can migrate into the blood circulation, which is consistent with previous reports.^9,35^ Similarly, we detect an increased number of Akk in blood circulation in the Akk-gavaged group, compared with the control. However, this is a temporary increase of Akk in blood circulation which appeared at 2 h after Akk gavage and then may be cleared after 6 h. Thus, we speculate that gut-derived Akk may migrate into blood circulation and subsequently colonize in tumor tissue before they are cleared in blood stream.

Several lines of evidence suggest that bacteria can be detected in the circulating system and they can probably survive there.^42,47,48^ The sources for these circulating bacteria could vary. In addition to gut, other sources may include oral cavity or skin or other organs. For example, Achromobacter is more likely derived from oral cavity, as suggested by this work based on comparison with databases of Human Microbiome Project (HMP).^49^ Oral bacteria such as Fusobacterium nucleatum can colonize into colorectal cancer via hematogenous spread, and lectin Fap2 is needed in this progression.^50^ Moreover, oral bacteria is the source of pancreatic microbiota, which is associated with pancreatic neoplasm.^51^ In addition, a close connection between gut microbiota and breast microbiota has been suggested.^52^ Gut fungi and bacteria also can invade into the TME of breast cancer via hematogenous spread.^53^ Thus, the circulatory system is a potential source of intratumoral microbes. In particular, systemic bacteria administration has been proven that bacteria can colonize into tumor microenvironment (TMEs) by hematogenous spread.^35^

Now a more interesting question is how these bacteria get into the circulating system. One possibility is that bacteria can regulate gut permeability through which they may be able to actively travel into the blood stream or may be passively leaked into blood stream. In this regard, Chelakkot et al.,^48^ showed that A. muciniphila influences gut permeability through the regulation of tight junctions, whereas circulating bacteria are usually derived from the gut, and increased permeability of intestinal wall due to gut vascular barrier disruption.^54^ Another possibility is that the intratumor microbes may go with the tumor cells especially when the tumor cells metastasize to distant locations through the blood stream where the microbes are released when the tumor cells are damaged and lysed. In terms of other mechanisms, characterized with abnormal and abundant vascular systems in tumor tissue, bacteria from the gut may translocate into tumor sites through bloodstream, and then enter tumor tissue by the damaged blood vessels.^55^ The necrotic cellular debris associated chemotactic gradient may also lead to the colonization of intratumor microbiota.^7^ Furthermore, high bacterial population levels may promote the translocation of certain bacteria from the gut to the mesenteric lymph nodes,^56^ and even blood circulation and distant organs. On the other hand, gut bacteria crossing the intestinal epithelial barrier may be through the extracellular vesicles produced by dendritic cells (DCs), which insert dendrites between the epithelial cells into the intestinal lumen. Finally, extracellular vesicles can enter the systemic circulation and be delivered to different organs.^57^

Of interest, live commensal bacteria can translocate to systemic tissues at steady state.^58^ There is a report that gut commensals can translocate to systemic sites in the presence of a microbiota complex, and in this case, both live E. coli and Lactobacillus acidophilus can be detected in systemic tissues upon their oral administration to the healthy SPF mice.^56^ Thus, we speculate that gut Akk may migrate into bloodstream through a similar mechanism. However, further studies are needed to define the mechanisms of gut bacteria (including Akk) translocation during homeostasis.

Our results demonstrated that tumor microbiota carry an increased number of Akkermansia or any other gut microbes in the Akk gavaged lung cancer mouse model, compared with the control, suggesting that gut-derived Akk may migrate into blood circulation, and subsequently colonize into lung cancer tissue. As a result, gavaged Akk may play a tumor suppressive role possibly through impacting the tumoral symbiotic microbiome and reprogramming tumoral metabolism. However, there are still more questions to be explored in future.

In summary, our results show that gut Akk could migrate and colonize into lung cancer tissue likely through blood circulation, and subsequently impact the symbiotic microbiome of lung cancer. Importantly, crosstalk of gut Akk with tumor microbiome may shape a favorable anticancer microenvironment by metabolic reprogramming, and thus, tumor microbiome may serve as a predictor of cancer outcomes. Furthermore, spatially resolved metabolomics analysis may serve as a new approach to explore the anticancer role of gut microbiome by regulating tumoral metabolism, and gain new insights into the understanding of gut microbiome-regulated cancer metabolic reprogramming at the molecular level, from metabolites to enzymes. Future work should further evaluate the cross-reactivity between gut bacteria (Akk)-regulated tumor microbiome and the metabolome in lung cancer.

Our study has several limitations. Due to the limited sample size of spatial metabolomics, the correlation between the differential microecology and metabolomics reported in this study needs further validation with more samples. In addition, given the limited resolution of microscopic images and the matched MS images, we are not able to tell whether tumor bacteria reside inside or outside of tumor cells. Finally, although our results suggest that gut-derived Akk inhibits lung cancer and impacts the symbiotic microecology in tumor tissue by direct interaction with tumors, gut Akk may also play the anticancer role through indirect ways, which needs further study in future.

## Methods

### Experimental model and study design

We evaluated the effects of gut endogenous Akk or gavaged exogenous Akk on tumorigenesis and proliferation using Lewis lung cancer mouse model. Mice were subject to subcutaneous injection with Lewis cancer cells, and then were monitored until sacrificed. Feces, blood, and tumor tissue samples for 16S rDNA sequencing were conducted to evaluate the microbiome composition and diversity. Tumor tissues were collected for spatially resolved metabolomics profiling to discover cancer metabolites and to characterize the overall gavaged Akk influenced tumor microbiota and metabolic features.

To evaluate the effects of endogenous gut Akk on tumorigenesis using Lewis lung cancer mouse model. A total of 108 C57BL/6 mice (6 to 8 weeks old) were kept in specific pathogen-free (SPF) conditions, and then each mouse was subject to a subcutaneous injection at right back with 1 × 10^6^ Lewis lung cancer cells in 100 ul sterile PBS. Mice were then divided into group 1 and group 2 based on tumor growth: mice with observable tumor nodules (4–5 mm diameter) appeared at 1 week after subcutaneous injection (named group 1); mice with observable tumor nodules (4–5 mm diameter) appeared at week 2 after subcutaneous injection (named group 2a) or did not show any tumorigenesis even at week 4 after subcutaneous injection (named group 2b). Mice were monitored until sacrificed, followed by proliferation evaluation. Fecal samples were collected for 16S rDNA sequencing to determine the composition and diversity of gut microbiota.

To determine the effects of exogenous gut Akk on tumorigenesis and proliferation, 24 C57BL/6 mice (6 to 8 weeks old) kept in SPF conditions were divided into three groups, i.e., control (con.), Akk and combined group (Akk/Bio). Akk/Bio mice were gavaged with both Akk and Bifidobacterium pseudolongum (Shanghai Guyan Industrial Co., Ltd). Akk or Bifidobacterium pseudolongum (2 × 10^8^ CFU in 0.15–0.2 ml PBS) was gavaged every 2 days, from week 2 before subcutaneous injection of 1 × 10^6^ Lewis cancer cells in 100 ul sterile PBS in each mouse. The control mice were gavaged with the same volume of PBS. The mice were kept under monitoring until they were sacrificed.

Feces, blood and tumor tissue samples of mice with or without Akk gavage were collected for 16S rDNA sequencing to dissect the fecal and tumoral microbiota, and to define the crosstalk between gut bacteria such as Akk with intratumoral microbiota.

Finally, tumor tissue samples from the control and the Akk gavaged lung cancer mouse model were collected. Each group contained four samples for the spatially resolved metabolomics profiling, to determine whether gut Akk is involved in the regulation of metabolic contexture and to characterize the overall metabolic features regulated by Akk, and to perform the correlation analysis regarding gut Akk-regulated intratumoral bacteria and metabolic network.

### Chemicals and reagents

MS-grade acetonitrile was purchased from Thermo Fisher (Thermo Fisher, U.S.A). Purified water was obtained from Watsons (Hongkong, China). Formic acid was provided by Merck (Merck, Germany), the tissue freezing medium was obtained from Leica (Leica Microsystem, Germany), Eosin Y-solution 0.5% aqueous and Hematoxylin was purchased from Sigma-Aldrich (St. Louis, MO, USA). Primary antibodies against LDHA (Cat.M00825-1) and GLS (Cat.A01272-2) were purchased from Boster Co., Ltd (Wuhan).

### Cell culture

The Lewis lung carcinoma (LLC) cells were provided by the cell bank of Chinese Academy of Sciences (Shanghai).^59^ The cells were tested for mycoplasma and the results were all negative. LLC cells were grown in DMEM (Hyclone) supplemented with 10% fetal bovine serum (Sigma-Aldrich) and 1% penicillin and streptomycin. The cells were incubated at 37°C and supplemented with 5% CO2 in the humidified chamber.

### Culture of Akkermansia muciniphila (Akk)

Akkermansia muciniphila (ATCC BAA-835) was provided by Biobw Biotech Co., Ltd (Beijing), and was cultured in brain heart infusion broth containing mucin, 10 mg/L resazurin (an oxidation-reduction indicator) under strict anaerobic conditions. A representative culture stock was used to determine the CFU/mL under anaerobic conditions by plate counting using mucin media containing 1% agarose. This culture was diluted with anaerobic PBS containing 2.5% glycerol to a final concentration of 1–2 × 10^9^ CFU in 200 µL.

### Mice

All animal procedures with all protocols receiving ethical evaluation were approved by the Institutional Animal Care and Use Committee (IACUC) of Tongji Hospital of Tongji University. All studies were performed in accordance with the NIH Guide for the Care and Use of Laboratory Animals. Male C57BL/6 mice at 6 ~ 8 weeks old purchased from Shanghai SLAC Laboratory Animal Co., Ltd. (China) were housed in a specific pathogen-free (SPF) condition, with free access to water and food.

### 16S rDNA sequencing

We used the method as described previously.^59^ DNA extraction and PCR amplification: Microbial DNA was extracted from mouse feces specimen using the E.Z.N.A.® Soil DNA Kit (Omega Bio-Tek, Norcross, GA, U.S.) according to the manufacturer’s protocols. The V4-V5 region of the bacteria 16S ribosomal RNA gene was amplified by PCR (95°C for 2 min, followed by 25 cycles at 95°C for 30s, 55°C for 30s, and 72°C for 30s and a final extension at 72°C for 5 min) using primers 515 F 5’-barcode- GTGCCAGCMGCCGCGG)-3’ and 907 R 5’-CCGTCAATTCMTTTRAGTTT-3’, where the barcode is an eight-base sequence unique to each sample. PCR reactions were performed in triplicate 20 μL mixture containing 4 μL of 5 × FastPfu Buffer, 2 μL of 2.5 mM dNTPs, 0.8 μL of each primer (5 μM), 0.4 μL of FastPfu Polymerase, and 10 ng of template DNA. Amplicons were extracted from 2% agarose gels and purified using the AxyPrep DNA Gel Extraction Kit (Axygen Biosciences, Union City, CA, U.S.) according to the manufacturer’s instructions and quantified using QuantiFluor™ -ST (Promega, U.S.). Library construction and sequencing as described previously:^59^ purified PCR products were quantified by Qubit®3.0 (Life Invitrogen) and every 24 amplicons whose barcodes were different were mixed equally. The pooled DNA product was used to construct Illumina Pair-End library following Illumina’s genomic DNA library preparation procedure. Then, the amplicon library was paired-end sequenced (2 × 250) on an Illumina HiSeq platform (Shanghai BIOZERON Co., Ltd) according to the standard protocols.

### Histology and immunohistochemistry (IHC)

Histological examination of tumor tissue was performed on 10% neutralized buffered formalin-fixed and paraffin-embedded tumor sections and stained for H&E staining as described previously,^59^ and was calculated and analyzed individually by a pathologist. The IHC characterization of LDHA, and GLS in tumor tissues are provided. For IHC assays, the paraffin sections were deparaffinized, endogenous enzymes were inactivated and antigens were thermally repaired. The sections were then blocked and stained with LDHA, GLS antibodies (Boster, Wuhan), followed by corresponding secondary antibody and a Streptavidin Biotin Complex kit (Boster, Wuhan). Stained slides were scanned by Pannoramic SCAN (3DHISTECH Kft, Budapest, Hungary). IHC was quantified by Image- Pro- Plus software and the mean density was determined based on the rate of integral optical density sum and area.

### Colonization with bacterial gavage

Akk for gavage inoculation were suspended in sterile, O2-free reduced PBS. Akk were resuspended in anaerobic PBS. C57BL/6 male mice were gavaged with Akk bacteria (2 × 10^9^ CFU in 0.2 ml PBS), every 2 days. Colonization of feces was monitored, and feces specimens and tumor tissue were collected during and at the end of the study. The control mice were gavaged with the same volume of sterilized PBS.

### Tissue sample preparation

The embedded samples were stored at −80°C before being sectioned. The samples were cut into consecutive sagittal slices 10 μm about 10 slices by a cryostat microtome (Leica CM 1950, Leica Microsystem, Germany) and were thaw-mounted on positive charge desorption plate (Thermo Scientific, U.S.A). Sections were stored at −80°C before further analysis. They were desiccated at −20°C for 1 h and then at room temperature for 2 h before MSI analysis. Meanwhile, an adjacent slice was left for hematoxylin-eosin (H&E) staining.

### Data acquisition and MSI analysis

The analyses were carried out with an AFADESI-MSI platform (Shanghai OE Biotech Co., Ltd., Shanghai, China) in tandem with a Q-Orbitrap mass spectrometer (Q Exactive, Thermo Scientific, U.S.A.). Here, the solvent formula was ACN/H2O (8:2) at negative mode and ACN/H2O (8:2, 0.1%FA) at positive mode and the solvent flow rate was 5 μL/min, the transporting gas flow rate was 45 L/min, the spray voltage was set at 7 kV, and the distance between the sample surface and the sprayer was 3 mm as was the distance from the sprayer to the ion transporting tube. The MS resolution was set at 70,000, the mass range was 70–1000 Da, the automated gain control (AGC) target was 2E6, the maximum injection time was set to 200 ms, the S-lens voltage was 55 V, and the capillary temperature was 350°C. The MSI experiment was carried out with a constant rate of 0.2 mm/s continuously scanning the surface of the tumor section in the x direction and a 100 μm vertical step in the y direction.

### Data processing

The collected .raw files were converted into .imML format and then imported into MSiReader (an open-source interface to view and analyze high resolving power MS imaging files on Matlab platform) for ion image reconstructions after background subtraction, region-specific MS profiles were precisely extracted by matching high-spatial resolution H&E images. The discriminating endogenous molecules of different tissue microregions were screened by a supervised statistical analytical method: OPLS-DA. Variable Importance of Projection (VIP) values obtained from the OPLS-DA model were used to rank the overall contribution of each variable to group discrimination. A two-tailed Student’s T-test was further used to verify whether the metabolites of difference between groups were significant. Differential metabolites were selected with VIP values greater than 1.0 and p-values less than 0.05.

### Analyte identification

FDR-controlled metabolite annotation for high-resolution imaging mass spectrometry and provides a reference implementation for False Discovery Rate-controlled metabolite annotation of high-resolution imaging mass spectrometry data. Then, the metabolites were performed high-resolution tandem MS directly from tissue sections.

### Statistical analysis

The microregions of pixels of lung cancer regions were selected, with histology-driven and characterized ion image overlays. A t-test was carried out to validate the significance of the discriminated metabolites between the two groups. A multitude of region-specific small molecule metabolites were screened and visualized in cancer tissues. All results were shown as the mean ± standard errors of the means (SEM). The t-test or chi-squared test were used for statistical analyses. Data were analyzed by GraphPad Prism soft-ware (Version 8.0, GraphPad Prism Software Inc., San Diego, CA), and a p-value <0.05 was considered significant.

## Data Availability

The 16S rDNA sequencing data have been deposited to the NCBI Sequence Read Archive (SRA) database (Accession Number: PRJNA846506. The SRA records can be accessible with the following link: https://www.ncbi.nlm.nih.gov/sra/PRJNA846506.). The dataset regarding the spatially resolved metabolomics profiling used and analyzed during the current study available from the corresponding author on reasonable request.
